# Prediction of intraventricular haemorrhage in preterm infants using time series analysis of blood pressure and respiratory signals

**DOI:** 10.1038/srep46538

**Published:** 2017-04-24

**Authors:** Jacqueline Huvanandana, Chinh Nguyen, Cindy Thamrin, Mark Tracy, Murray Hinder, Alistair L. McEwan

**Affiliations:** 1 School of Electrical and Information Engineering, University of Sydney, Sydney, Australia; 2 Woolcock Institute of Medical Research, University of Sydney, Sydney, Australia; 3 Westmead Hospital, Sydney, Australia; 4 School of Paediatrics and Child Health, University of Sydney, Sydney, Australia

## Abstract

Despite the decline in mortality rates of extremely preterm infants, intraventricular haemorrhage (IVH) remains common in survivors. The need for resuscitation and cardiorespiratory management, particularly within the first 24 hours of life, are important factors in the incidence and timing of IVH. Variability analyses of heart rate and blood pressure data has demonstrated potential approaches to predictive monitoring. In this study, we investigated the early identification of infants at a high risk of developing IVH, using time series analysis of blood pressure and respiratory data. We also explore approaches to improving model performance, such as the inclusion of multiple variables and signal pre-processing to enhance the results from detrended fluctuation analysis. Of the models we evaluated, the highest area under receiver-operator characteristic curve (5th, 95th percentile) achieved was 0.921 (0.82, 1.00) by mean diastolic blood pressure and the long-term scaling exponent of pulse interval (PI *α*_2_), exhibiting a sensitivity of >90% at a specificity of 75%. Following evaluation in a larger population, our approach may be useful in predictive monitoring to identify infants at high risk of developing IVH, offering caregivers more time to adjust intensive care treatment.

Intraventricular Haemorrhage (IVH) remains a serious threat to survival for preterm infants and neurodevelopmental outcomes^1^. Despite advances in modern neonatal care such as antenatal steroids, artificial surfactant treatment and the use of neuroprotective agents such as magnesium sulphate given to mothers in labour, rates of IVH, particularly high grade, remain unchanged. Prematurity, respiratory-distress syndrome and mechanical ventilation are among the factors that may predispose infants to IVH. Recent studies have also suggested an association between IVH and cerebral pressure passivity, that is, where changes in cerebral blood flow correspond to changes in blood pressure^2^. The need for resuscitation and cardiorespiratory management of preterm infants within the first 24 hours of life play an important role in the development and timing of IVH^3^,^4^, where the majority of these cases can be detected at their full extent by the end of the first postnatal week^5^. The potential to identify infants at high risk of developing IVH is thus, particularly important.

Retrospective studies of premature infants after the diagnosis of IVH have highlighted altered autonomic functions which are reflected by heart rate variability analysis^6^,^7^. In particular, one study showed that these differences could be detected using electrocardiogram data from the first 24 hours of life^8^. Variability of beat-to-beat systolic blood pressure and mean arterial pressure has also been shown to offer useful information in distinguishing infants who later developed IVH from those who did not^9^. Such distinctions were demonstrated using detrended fluctuation analysis (DFA), a non-linear time domain technique that is able to quantify long-range power law correlations in a given time series. Its application is characterised by a scaling exponent (*α*) which can be calculated over different time scales and indicates the corresponding degree of correlation^10^,^11^.

More recent work in this area by Fairchild *et al*. has demonstrated associations between a heart rate characteristic index and adverse neurodevelopmental outcomes or white matter damage^12^. Models for early prediction of IVH have explored either clinical risk factors, as in the case of Luque *et al*.^13^ with an AUC of 0.79, or employed time series analysis techniques and physiological signals as in Tuzcu *et al*. who reported a sensitivity of 70% and specificity of 79% for their model using heart rate variability^8^.

The objective of this study was twofold; firstly, to explore means of improving prediction of IVH from DFA through pre-processing, and secondly, to evaluate the potential of multivariable or multimodal models in the prediction of IVH. The latter objective focused specifically on combinations of blood pressure and respiratory features and inherently involved an evaluation of robustness as applied in a clinical context. The features evaluated comprised of the mean (*μ*) as well as short- and long-term scaling exponents derived from DFA (*α*_1_ and *α*_2_, respectively), extracted from five different time series. These were: mean arterial pressure (MAP), systolic blood pressure (SBP), diastolic blood pressure (DBP) and pulse interval (PI) series as derived from the arterial blood pressure data, as well as the interbreath intervals (IBI) from the respiratory air flow data.

## Results

### Study Population

The study cohort consisted of 27 low birth-weight (<1500 g) infants, 8 of which subsequently developed IVH. We examined the differences in physiological characteristics and other metadata between the two groups, as summarised in Table 1. Although the IVH and non-IVH groups did not exhibit significant differences in the collected metadata, certain clinical characteristics, namely, the mean DBP values were observed to be significantly different for the two groups after non-linear trend removal (*p* < 0.05). This established a foundation for fitting the univariate logistic regression models, though evaluation of their overall performance requires reference to leave-one-out cross-validation (LOOCV) results.

### Effect of Detrending

**Figure 1:**
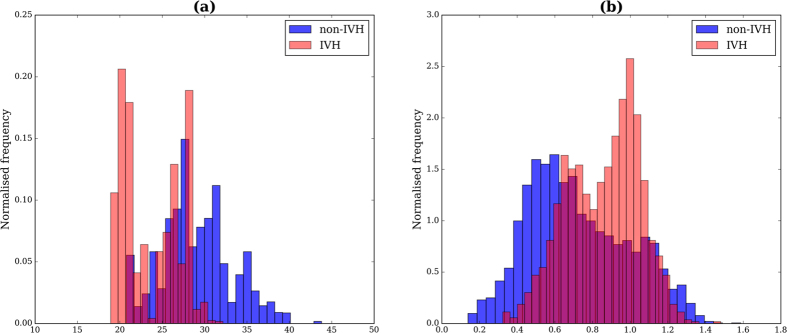
Normalised histograms of (**a**) mean DBP and (**b**) DBP *α*_1_ for IVH and non-IVH groups. The distributions for each group were based on features extracted from all individual windows which met the quality criteria.

In the initial stages of model fitting, we observed that in certain instances, the arterial blood pressure was subject to non-linear drift. We examined the effect of detrending the arterial blood pressure signal and noted that prediction performance of the fitted logistic regression models could be improved. This detrending affected the linear blood pressure features in particular, which also propagated to the beat-to-beat blood pressure values and thus the scaling exponents derived from DFA (*α*_1_ and *α*_2_, the short- and long-term scaling exponents, respectively). The Mann-Whitney U-test comparisons of the non-detrended features are shown in Table 2, where the effect of detrending was characterised by the changes in AUC and *p* values from the two-sided Mann-Whitney U-test. For example, the AUC scores of the mean DBP model improved from 0.757 to 0.807 subsequent to detrending. A similar increase from 0.757 to 0.771 was also observed for the univariate model of the long-term scaling exponent of DBP (DBP *α*_2_), motivating the inclusion of this pre-processing step for the subsequent analyses. The histograms of mean DBP and DBP *α*_1_ are also shown in Fig. 1.

### Univariate Predictors of IVH

We fitted univariate logistic regression models using various linear and DFA features, taking the mean across all qualifying time windows of data for each subject. We evaluated the AUC, the 95% confidence interval (5th, 95th percentile) according to the Delong method for determining standard error^14^, as well as the positive likelihood ratio and threshold corresponding to a specificity of 75%. These results are summarised in Table 3. Overall, the short-term fractal exponents (*α*_1_) derived from the MAP, SBP and DBP signals as well as the mean DBP yielded the highest AUCs. Respiratory variables were not found to be strongly predictive.

### Multivariable Predictors of IVH

It was also observed that model performance could also be improved through combination of the predictors extracted for the univariate models, as shown in Table 4. Predictor combinations that were significantly correlated were excluded to mitigate effects of collinearity. Many of the multivariable models exhibited higher AUC scores than univariate models, with the highest being the combination of mean DBP and PI *α*_2_.

**Figure 2:**
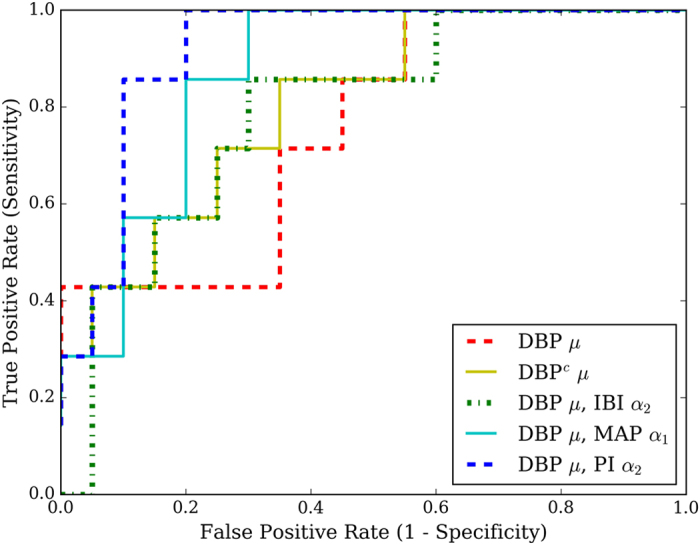
Receiver-Operator Characteristic Curves. These show the ROC curves for the non-detrended univariate mean DBP (DBP *μ*) model, the impact of detrending this feature (DBP^*c*^ *μ*), the addition of a respiratory feature (IBI *α*_2_) as well as two of the highest-scoring models (DBP *μ* combined with MAP *α*_1_ and PI *α*_2_, respectively).

**Figure 3:**
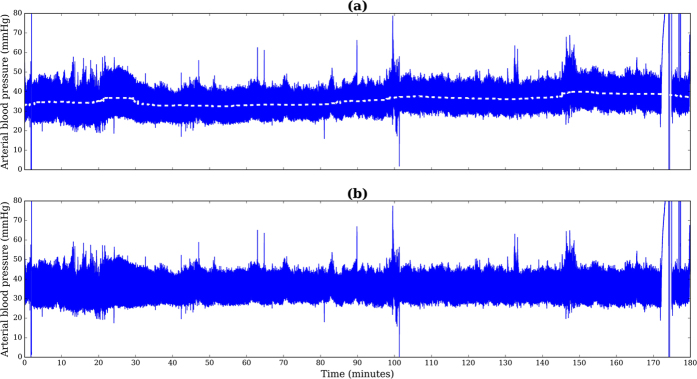
Detrending of overall segment. (**a**) shows the original signal and the corresponding non-linear trend, while (**b**) displays the signal after removal of this trend.

Similar results were obtained when evaluating all qualifying windows and with the inclusion of gestational age. Note however that the comparisons with the best performing univariate model (i.e. mean DBP, Table 3) were not statistically significant (*p* > 0.05) due to high degree of overlap. The ROC curves for a number of these models are displayed in Fig. 2, where the non-linear detrending process to obtain the DBP^*c*^ *μ* feature is illustrated in Fig. 3.

### Leave-One-Out Cross-Validation

The models were further evaluated using LOOCV, where the probability estimates of each testing sample were used to construct a receiver-operator characteristic (ROC) curve^15^,^16^, as summarised in the latter column of Table 4. Delong comparison of these LOOCV ROC curves with the corresponding LOOCV mean DBP model did not exhibit statistically-significant results (*p* > 0.05), with the exception of the mean DBP and SBP *α*_1_ combination.

### Sensitivity Analysis

The models used to obtain these results were based on the mean feature(s) calculated from all qualifying 10 min windows for each subject, with a 30 sec overlap. To evaluate how robust these results were, we examined the effect of including all individual windows rather than the mean feature per subject, of using non-overlapping windows and of including gestational age. The first two cases involved use of a mixed-model allowing for repeated measures while the latter involved the addition of gestational age as a predictor in the existing multivariable models. In all three cases, we found that mean DBP and PI *α*_2_ remained the most predictive combination for IVH (where AUC = 0.88, 0.86 and 0.85 for the three cases, respectively, compared to the AUC = 0.92 reported in Table 4).

## Discussion

### Summary of findings

This study evaluated the blood pressure and respiratory features in discerning infants with IVH from those without. The highest AUC achieved was 0.921 (95% CI 0.82, 1.00) by the model fitted with PI *α*_2_ and mean DBP. The results of cross-validation also supported this, with an AUC_*LOOCV*_ of 0.821 (0.66, 0.99). This model exhibited a sensitivity of >90% at a specificity of 75% which is greater than that reported for the heart rate variability-based model from Tuzcu *et al*. (70% sensitivity, 79% specificity)^8^. This latter cohort was of a similar size (n = 24), though it was limited to very low birthweight infants (<1000 g) as opposed to our low birthweight cohort, potentially contributing to the difference in IVH representation observed (41.7% compared to 29.6%). Although the univariate model fitted with mean DBP exhibited an AUC of 0.807 in the initial analysis, results from LOOCV cautioned its use as a sole predictor, with an AUC of 0.607 and a non-significant 95% confidence interval of (0.38, 0.88).

### Effect of detrending

Out of all the factors we examined, detrending as part of the pre-processing phase of analysis resulted in the greatest improvement in prediction of IVH. It rendered both mean DBP and DBP *α*_2_ significantly different (*p* < 0.05) between the IVH and non-IVH groups, where the AUC scores for two univariate models fitted with these features increased from 0.757 to 0.807 and 0.771, respectively (Tables 2 and 3). These improvements suggest that long-term drift and/or baseline wander of the blood pressure signals, among other aspects of signal quality, may confound the results from DFA as well as linear parameters. The significant contribution of mean DBP is of particular importance, given previous assertions that linear features alone were not an informative proxy of cerebral perfusion pressure^17^ and reports that such features were not significantly different between the IVH and non-IVH groups^9^. This relationship to DBP may also have been a reflection of a widened pulse pressure, seen in symptomatic patent ductus arteriosus. It is necessary to note however that this feature was not explicitly evaluated in the previous studies. Aside from the detrending, this analysis differed in other aspects including the quality control measures for window selection and the evaluation process; the features extracted represent the mean of all qualifying 10 min windows across the recording, rather than a single segment. In the clinical application of DFA to monitored signals, we strongly recommend examination of signal data to determine whether overall detrending is necessary prior to analysis.

### Use of bivariate models

Subsequent to detrending, a further increase in AUC achieved through fitting of bivariate models, where the combination of mean DBP with MAP *α*_1_ and PI *α*_2_, for example, exhibited respective scores of 0.871 and 0.921. This would suggest that relevant, non-redundant information may be captured using linear and DFA-based approaches in the prediction of IVH. The short-term scaling exponents for the beat-to-beat MAP and SBP, along with mean DBP were shown to be relatively robust markers in prediction of IVH for the studied cohort. Although all of the studied infants had triggered ventilation modes (synchronised intermittent positive pressure ventilation), breathing termination was not employed and so the potential for adverse patient ventilator interaction was possible. Previous work examining the impact of patient-ventilator asynchrony indicates the significant potential for IVH with this phenomenon^18^,^19^. These results align with those previously reported^9^ and the altered vagal nerve activity in infants with IVH^20^. From further evaluation of the bivariate models, it was clear that the initial analysis did not necessarily translate to robust and consistent performance in leave-one-out cross-validation. The model with the highest AUC in the initial analysis achieved an AUC_*LOOCV*_ of 0.821 (0.66, 0.99), though it did not exhibit a statistically significant improvement on the univariate reference model (mean DBP)^14^. It was interesting to note the inclusion of pulse interval-based features in the highest-scoring model in both the initial analysis and cross-validation, given its use as an estimate of heart rate variability and the reported high correlation between the two^21^,^22^. The accuracy of this estimation however, has not been clarified, particularly in the neonatal context, though electrocardiogram-based heart rate variability has been found to offer useful information in distinguishing infants with and without IVH^8^.

### Addition of respiratory signals

In this study, we found that the addition of respiratory signals did not considerably improve model performance. The fractal dynamics of respiration have been applied in the context of preterm infants^23^,^24^, though not with respect to IVH. As for the models fitted with interbreath interval (IBI) features, mechanical ventilation may have contributed to their observed lack of prediction capacity (*p* > 0.05), despite the relevance of respiration mechanics in the development of IVH^18^. It is also possible that the IBI-based features were not suited to characterising patient-ventilator asynchrony.

### Clinical significance and application

Hypercarbia, high ventilator pressure and patency of the ductus arteriosus are among the factors and events that may influence the fluctuation of blood pressure of preterm infants in the neonatal intensive care unit^25^. Infants who later developed IVH exhibited lower mean DBP and a higher DBP *α*_2_ (*p* < 0.05) across the entire recording in this study. Recent studies have reported a range of observations pertaining to blood pressure and IVH, with the main focus on characterising cerebral perfusion. These include reports of IVH being associated with the elevated diastolic closing margin^17^ and significant deviation above a defined optimal MAP value in infants who later developed IVH^26^.

**Figure 4:**
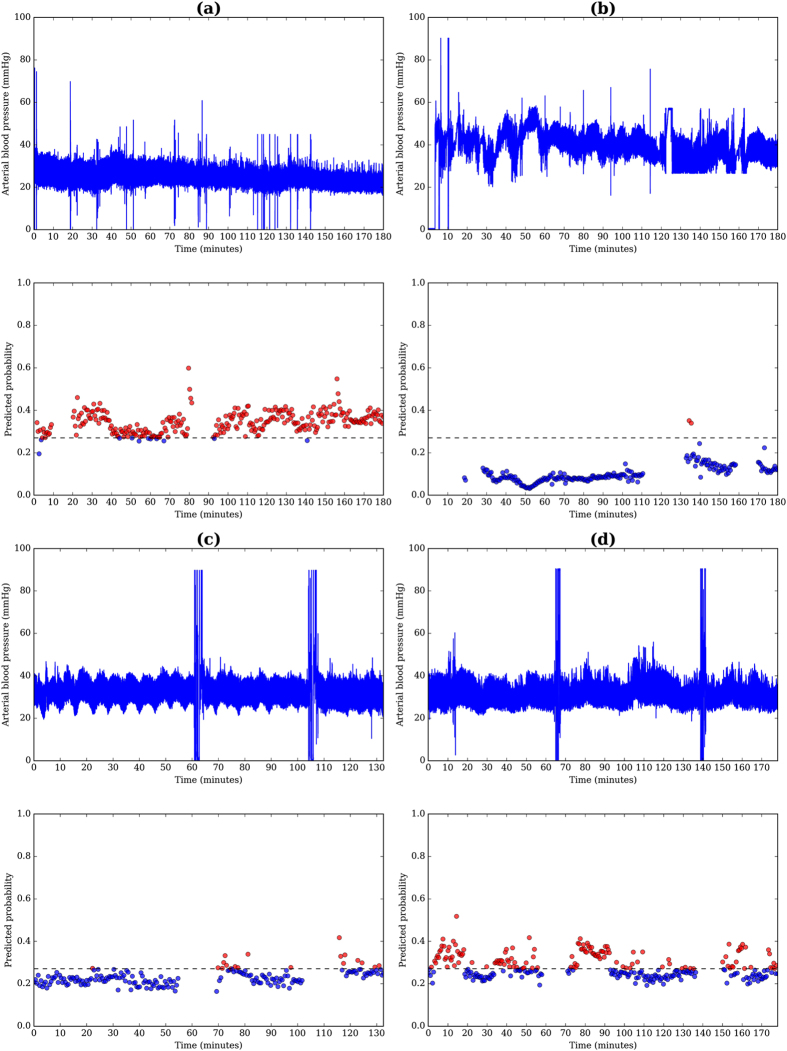
Arterial blood pressure data and the predicted probability for IVH using the highest scoring model, mean DBP and PI *α*_2_ for correct classification of (**a**) IVH and (**b**) non-IVH, as well as incorrect classification of (**c**) IVH and (**d**) non-IVH. The threshold for classifying IVH, designated by the dashed line was defined at 90% specificity and 85% sensitivity. Red and blue markers represent windows that exceeded and did not exceed the threshold, respectively.

This approach may be applied to a clinical context in a manner similar to that shown in Fig. 4, offering examples of both correct and incorrect classification of IVH from the studied cohort. A threshold may be defined according to the dashed line in each of the cases (a) to (d), where calculated probabilities exceeding this threshold could flag infants at high-risk of developing IVH. Further model evaluation requires validation on a larger and more balanced cohort to estimate the prediction error and support its potential application in a clinical context.

### Limitations

We acknowledge that this study was limited by the size of the dataset (n = 27) as well as representation of IVH (29.6%), slightly lower than the referenced 35–45% of incidence reported in neonatal care facilities^27^. Model evaluation was also limited by the low number of IVH cases (n = 8), though our LOOCV and sensitivity analyses showed the main findings to be consistent. Another limiting factor was the signal quality of the recordings which was managed by implementing quality control measures as part of the feature extraction process.

## Conclusion

In conclusion, this study found mean DBP and short-term scaling exponents from beat-to-beat MAP, DBP and SBP to be useful markers in the prediction of IVH in preterm infants. Non-linear trend removal and the inclusion of additional features such as the short-term scaling exponent (*α*_1_) of MAP was able to improve model performance. Of the models evaluated, the one that performed consistently in both the initial analysis and cross-validation was fitted with mean DBP and PI *α*_2_. In a clinical context, such an approach to signal processing and predictive monitoring could be applied, where a running 10 min window could continuously evaluate the relevant features from qualifying segments of data. Following evaluation in a larger population, these features may be helpful in identifying infants at high-risk of developing IVH, offering caregivers more time to adjust intensive care treatment.

## Methods

### Data Collection

Physiological data was collected from the infants within 1–3 hours of birth as part of a prospective clinical investigation at a large tertiary neonatal intensive care unit in Sydney, Australia. The study was approved by the Sydney West Area Health Service Human Research and Ethics and conducted according to the World Medical Association Declaration of Helsinki. Informed parental consent was obtained in all cases.

Inclusion criteria for the cohort comprised low birthweight (<1500 g), gestational age (<30 weeks) and an absence of significant congenital anomalies. Of the 46 infants enrolled, 27 infants had arterial blood pressure and air flow wave recordings with sufficiently long, artefact-free segments. The average (SD) length of recording was 156 (34) mins. Intra-arterial blood pressure was measured using an umbilical or peripheral arterial catheter, following single-point calibration to atmospheric pressure, collected using a bedside patient monitor (Philips Agilent Systems, Philip Healthcare, North Ryde, Australia), while the raw air flow wave was acquired from a ventilator (Babylog 8000, Drägerwerk, Lübeck, Germany). Both signals were sampled at 1 kHz and recorded by an analog data acquisition system (ADInstruments, Sydney, Australia). Cranial ultrasounds were performed at 2, 12, 24 and 36 hours then daily for the first week. The presence and grade of IVH was determined according to the Papile system^27^.

### Signal Processing and Data Analysis

Signal processing and feature extraction was completed in Python (Python Software Foundation, version 2.7. https://www.python.org/). Each of the arterial blood pressure and air flow signals were down-sampled to 125 Hz prior to analysis for computational efficiency. This frequency was sufficient for peak detection in both respiratory and blood pressure signals. From the downsampled signals, the following time series were extracted; the beat-to-beat MAP, SBP, DBP and PI, as derived from arterial blood pressure, as well as IBIs derived from air flow data. The signal quality constraints of the air flow data limited extraction of other respiratory features such as peak flow.

Only the arterial blood pressure signal was found to exhibit significant drift, defined by non-linear trends in the diastolic and systolic blood pressure ranges. Thus, the detrending was applied solely to this signal, as shown in Fig. 3. Such a correction would also minimally impact the derivation of the IBI-based features from the air flow signal. The overall trend of each signal was determined using a large-window median filter (window width = 1000 ms) on a further downsampled signal and the mean-centred trend was subsequently removed from the original arterial blood pressure signal. This approach was similar to the baseline wander removal that has been applied widely prior to feature extraction from the electrocardiogram signal^28^. An example of this detrending process is shown in Fig. 3.

The features used in IVH prediction were extracted from a running 10 min window of arterial blood pressure and air flow data, shifted in 30 sec increments across the total recording length. This approach was adopted to simulate the application of these techniques in a clinical setting, where windows which fulfilled the quality criteria were included for feature extraction. This criteria comprised defined ranges for the allowable number of beats and breaths in a given window (40–250 beats per minute and >20 breaths per minute), a maximum limit for an absence of detected beats (15 sec) as well as an absence of large spikes in the arterial blood pressure signal (range of beat-to-beat SBP <30 mmHg). For each respective time series, outliers were removed by imposing a maximum change of 150% from the previous data point and also a maximum loss of 30% for each window. Features including the mean (*μ*), short- and long-term scaling exponents (*α*_1_, *α*_2_, respectively) from DFA of the five time series (MAP, SBP, DBP, PI and IBI) were subsequently extracted.

Developed by Peng and co-workers^10^, DFA is able to quantify long-range power law correlations and accommodate for confounding non-stationarities often seen in real-world signals. It does this through the detrending, that is, linear trend removal, step prior to calculating the root-mean squared fluctuation as defined in equation 1.


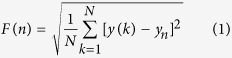


where *y*(*k*) is any given time series, *y*_*n*_(*k*) the local linear trend for a given segment, and *N* the number of data points in the series for a given round of analysis. The application of DFA is further explained by Thamrin *et al*.^11^.

The scaling exponent *α* is calculated from the gradient of the line fitted to the Log-Log relationship between n and F(n). In this case, the short-term scaling exponent was defined over 4–15 beats, as aligned with similar observations of this data^9^ and similarly defined for heart rate variability analysis of preterm infants^8^. The long-term scaling exponent was determined across 15–50 beats.

### Model Fitting and Evaluation

Statistical analysis was completed using R 3.3.1 software^29^. Logistic regression models were used to fit the mean of extracted predictors across all qualifying 10 min windows, while the AUC was used to assess accuracy in predicting IVH. Fitted models were evaluated using leave-one-out cross-validation, where the predicted probability of each test sample was subsequently compiled and used to generate a ROC curve for performance comparison.

## Additional Information

**How to cite this article**: Huvanandana, J. *et al*. Prediction of intraventricular haemorrhage in preterm infants using time series analysis of blood pressure and respiratory signals. *Sci. Rep.* **7**, 46538; doi: 10.1038/srep46538 (2017).

**Publisher's note:** Springer Nature remains neutral with regard to jurisdictional claims in published maps and institutional affiliations.

## Figures and Tables

**Table 1:** Comparison of physiological variables between infants who later developed intraventricular haemorrhage (IVH) and those who did not (non-IVH).

Variable	IVH (n = 7)	Non-IVH (n = 20)	*p*
Gestational age (weeks)	26.8 ± 1.2	26.9 ± 1.8	0.781
Birthweight (g)	1120 ± 282	1029 ± 293	0.580
Sex (% male)	57.1 ± 49.5	65.0 ± 47.7	0.741
CRIBII	9 ± 1	9 ± 2	1.000
PDA (%)	85.7 ± 35.0	80.0 ± 40.0	0.774
RDS	1.0 ± 0.0	1.0 ± 0.0	1.000
Apgar 1-min	6 ± 1	6 ± 2	0.696
Apgar 5-min	7 ± 1	7 ± 1	0.421
MAP (mmHg)	32.5 ± 6.1	35.2 ± 4.7	0.422
DBP (mmHg)	25.0 ± 3.9	29.0 ± 4.6	0.050
MAP^c^ (mmHg)	32.1 ± 5.6	35.5 ± 4.2	0.234
DBP^c^ (mmHg)	24.6 ± 3.5	29.4 ± 4.1	0.019

Values are reported as mean ± SD. ^c^Denotes detrended features, CRIBII is the Clinical Risk Index for Babies score II, PDA is Patent Ductus Arteriosus and RDS is Respiratory Distress Syndrome. *p* values are derived from a two-sided Mann-Whitney U-test where significance is defined as *p* < 0.05.

**Table 2:** Effect of Detrending.

Variable	IVH	Non-IVH	AUC	AUC^*c*^	*p*	*p*^*c*^
MAP						
*μ*	32.5 ± 6.1 mmHg	35.2 ± 4.7 mmHg	0.607	0.657	0.422	0.234
*α*_1_	0.96 ± 0.17	0.78 ± 0.19	0.779	0.779	0.033	0.033
*α*_2_	1.10 ± 0.06	1.00 ± 0.18	0.671	0.65	0.194	0.257
SBP						
*μ*	41.9 ± 9.7 mmHg	42.7 ± 5.4 mmHg	0.564	0.55	0.638	0.719
*α*_1_	0.83 ± 0.11	0.69 ± 0.15	0.764	0.771	0.043	0.038
*α*_2_	1.04 ± 0.08	0.97 ± 0.16	0.643	0.664	0.281	0.213
DBP						
*μ*	25.0 ± 3.9 mmHg	29.0 ± 4.6 mmHg	0.757	0.807	0.05	0.019
*α*_1_	0.85 ± 0.12	0.68 ± 0.20	0.786	0.807	0.029	0.019
*α*_2_	1.05 ± 0.06	0.93 ± 0.16	0.757	0.771	0.05	0.038

Values are reported as mean ± SD, p values are from Mann-Whitney U-tests from the non-detrended data. AUC is the area under the ROC curve for prediction of IVH. Note that AUC^*c*^ and p^*c*^ are obtained from the detrended data.

**Table 3:** Univariate Logistic Regression models.

Model	AUC (95% CI)	*p*	Threshold	LR
MAP				
*μ*	0.657 (0.37, 0.95)	0.218	31.72 mmHg	2.29
*α*_1_	0.779 (0.60, 0.96)	0.359	0.92	2.86
*α*_2_	0.650 (0.44, 0.86)	0.839	1.08	2.40
SBP				
*μ*	0.550 (0.20, 0.90)	0.389	37.96 mmHg	2.29
*α*_1_	0.771 (0.58, 0.96)	0.382	0.81	2.86
*α*_2_	0.664 (0.43, 0.90)	0.792	0.94	1.60
DBP				
*μ*	0.807 (0.62, 0.99)	0.022	26.34 mmHg	2.86
*α*_1_	0.807 (0.64, 0.97)	0.278	0.79	3.43
*α*_2_	0.771 (0.59, 0.95)	0.415	1.02	2.80
PI				
*μ*	0.543 (0.25, 0.83)	0.759	50.10 ms	1.40
*α*_1_	0.607 (0.38, 0.84)	1.000	0.42	1.40
*α*_2_	0.707 (0.45, 0.97)	0.709	1.08	2.29
IBI				
*μ*	0.707 (0.46, 0.96)	0.643	115.88 ms	2.40
*α*_1_	0.500 (0.25, 0.75)	0.568	0.52	1.14
*α*_2_	0.557 (0.26, 0.85)	0.813	0.45	0.40

Models were fitted with mean (*μ*), short- and long-term scaling exponents (*α*_1_ and *α*_2_, respectively) for five time series: mean arterial (MAP), systolic (SBP) and diastolic (DBP) blood pressure, as well as pulse (PI) and interbreath (IBI) intervals. Positive likelihood ratios (LR) and corresponding thresholds are reported at a specificity of 75%. 95% confidence intervals (CI) and *p* values reported for the AUC are derived from the Delong approach^14^ for determining standard error and comparison with the reference ROC of the non-detrended mean MAP model.

**Table 4:** Multivariable logistic regression models.

Feature 1	Feature 2	AUC (95% CI)	*p*	LR	AUC_*LOOCV*_
SBP *α*_1_	DBP *μ*	0.843 (0.68, 1.01)	0.009	2.86	0.721 (0.50, 0.94)*
PI *α*_1_	DBP *μ*	0.843 (0.69, 1.00)	0.014	2.86	0.643 (0.40, 0.89)
DBP *α*_1_	DBP *μ*	0.864 (0.72, 1.00)	0.022	2.86	0.750 (0.56, 0.94)
PI *α*_2_	MAP *μ*	0.864 (0.72, 1.01)	0.068	3.43	0.679 (0.46, 0.89)
MAP *α*_1_	DBP *μ*	0.871 (0.74, 1.01)	0.027	3.43	0.743 (0.55, 0.94)
PI *α*_2_	DBP *μ*	0.921 (0.82, 1.02)	0.035	4.00	0.821 (0.66, 0.99)

Features included mean (*μ*), short- and long-term scaling exponents (*α*_1_ and *α*_2_, respectively) for mean arterial (MAP), systolic (SBP) and diastolic (DBP) blood pressure, as well as pulse interval (PI) time series. LR denotes the positive likelihood ratios, the 95% confidence intervals (CI) are reported for the AUC. *p* values are derived from the Delong comparison^14^ with the non-detrended mean MAP model. The corresponding AUCs from leave-one-out cross-validation (AUC_*LOOCV*_) are also reported, where *denotes a statistically-significant (*p* < 0.05) difference from the Delong comparison of the LOOCV mean DBP model^14^.

## References

[b1] Bolisetty S. . Intraventricular hemorrhage and neurodevelopmental outcomes in extreme preterm infants. Pediatrics peds –2013 (2013). 10.1542/peds.2013-0372 24379238

[b2] O’Leary H. . Elevated cerebral pressure passivity is associated with prematurity-related intracranial hemorrhage. Pediatrics 124, 302–309 (2009). 19564313 10.1542/peds.2008-2004 PMC4030537

[b3] Fanaroff A. A. . Trends in neonatal morbidity and mortality for very low birthweight infants. American journal of obstetrics and gynecology 196, 147–e1 (2007). 17306659 10.1016/j.ajog.2006.09.014

[b4] Landmann E., Misselwitz B., Steiss J. O. & Gortner L. Mortality and morbidity of neonates born at less than 26 weeks of gestation (1998–2003). A population-based study. Journal of perinatal medicine 36, 168–174 (2008). 18257656 10.1515/JPM.2008.016

[b5] Ment L. R. . Risk factors for early intraventricular hemorrhage in low birth weight infants. The Journal of pediatrics 121, 776–783 (1992). 1432433 10.1016/s0022-3476(05)81915-8

[b6] van Ravenswaaij-Arts C. M. . The influence of respiratory distress syndrome on heart rate variability in very preterm infants. Early human development 27, 207–221 (1991). 1802672 10.1016/0378-3782(91)90195-9

[b7] Hanna B. . Heart rate variability in preterm brain-injured and very-low-birth-weight infants. Neonatology 77, 147–155 (2000). 10.1159/000014209 10729717

[b8] Tuzcu V., Nas S., Ulusar U., Ugur A. & Kaiser J. R. Altered heart rhythm dynamics in very low birth weight infants with impending intraventricular hemorrhage. Pediatrics 123, 810–815 (2009). 19255007 10.1542/peds.2008-0253 PMC2871543

[b9] Zhang Y. . Detrended fluctuation analysis of blood pressure in preterm infants with intraventricular hemorrhage. Medical & biological engineering & computing 51, 1051–1057 (2013). 23716182 10.1007/s11517-013-1083-0

[b10] Peng C.-K., Havlin S., Stanley H. E. & Goldberger A. L. Quantification of scaling exponents and crossover phenomena in nonstationary heartbeat time series. Chaos: An Interdisciplinary Journal of Nonlinear Science 5, 82–87 (1995). 10.1063/1.166141 11538314

[b11] Thamrin C. & Stern G. New methods: what do they tell us? Fluctuation analysis of lung function. Eur Respir Mon 47, 310–324 (2010).

[b12] Fairchild K. . Abnormal heart rate characteristics are associated with abnormal neuroimaging and outcomes in extremely low birth weight infants. Journal of Perinatology 34, 375–379 (2014). 24556979 10.1038/jp.2014.18 PMC11019753

[b13] Luque M. . A risk prediction model for severe intraventricular hemorrhage in very low birth weight infants and the effect of prophylactic indomethacin. Journal of Perinatology 34, 43–48 (2014). 24113396 10.1038/jp.2013.127

[b14] DeLong E. R., DeLong D. M. & Clarke-Pearson D. L. Comparing the areas under two or more correlated receiver operating characteristic curves: a nonparametric approach. Biometrics 837–845 (1988). 3203132

[b15] LeDell E., Petersen M. & van der Laan M. Computationally efficient confidence intervals for cross-validated area under the roc curve estimates. Electronic journal of statistics 9, 1583 (2015). 26279737 10.1214/15-EJS1035 PMC4533123

[b16] Gönen M. Analyzing receiver operating characteristic curves with SAS (SAS Institute, 2007).

[b17] Rhee C. J. . Elevated diastolic closing margin is associated with intraventricular hemorrhage in premature infants. The Journal of pediatrics (2016). 10.1016/j.jpeds.2016.03.066 PMC4925245 27112042

[b18] Perlman J. M., McMenamin J. B. & Volpe J. J. Fluctuating cerebral blood-flow velocity in respiratory distress syndrome: relation to the development of intraventricular hemorrhage. New England Journal of Medicine 309, 204–209 (1983). 6866033 10.1056/NEJM198307283090402

[b19] Perlman J. M., Goodman S., Kreusser K. L. & Volpe J. J. Reduction in intraventricular hemorrhage by elimination of fluctuating cerebral blood-flow velocity in preterm infants with respiratory distress syndrome. New England Journal of Medicine 312, 1353–1357 (1985). 3887165 10.1056/NEJM198505233122104

[b20] Koketsu N., Moskowitz M. A., Kontos H. A., Yokota M. & Shimizu T. Chronic parasympathetic sectioning decreases regional cerebral blood flow during hemorrhagic hypotension and increases infarct size after middle cerebral artery occlusion in spontaneously hypertensive rats. Journal of Cerebral Blood Flow & Metabolism 12, 613–620 (1992). 1618940 10.1038/jcbfm.1992.85

[b21] Schäfer A. & Vagedes J. How accurate is pulse rate variability as an estimate of heart rate variability?: A review on studies comparing photoplethysmographic technology with an electrocardiogram. International journal of cardiology 166, 15–29 (2013). 22809539 10.1016/j.ijcard.2012.03.119

[b22] Bulte C. S., Keet S. W., Boer C. & Bouwman R. A. Level of agreement between heart rate variability and pulse rate variability in healthy individuals. European Journal of Anaesthesiology (EJA) 28, 34–38 (2011). 10.1097/EJA.0b013e32834088c4 20962650

[b23] Baldwin D. N. . Effect of sighs on breathing memory and dynamics in healthy infants. Journal of Applied Physiology 97, 1830–1839 (2004). 15208293 10.1152/japplphysiol.00298.2004

[b24] Frey U., Silverman M., Barabasi A. & Suki B. Irregularities and power law distributions in the breathing pattern in preterm and term infants. Journal of Applied Physiology 85, 789–797 (1998). 9729549 10.1152/jappl.1998.85.3.789

[b25] Ballabh P. Intraventricular hemorrhage in premature infants: mechanism of disease. Pediatric research 67, 1–8 (2010). 19816235 10.1203/PDR.0b013e3181c1b176 PMC2799187

[b26] da Costa C. S. . Monitoring of cerebrovascular reactivity for determination of optimal blood pressure in preterm infants. The Journal of pediatrics 167, 86–91 (2015). 25891381 10.1016/j.jpeds.2015.03.041

[b27] Papile L.-A., Burstein J., Burstein R. & Koffler H. Incidence and evolution of subependymal and intraventricular hemorrhage: a study of infants with birth weights less than 1,500 gm. The Journal of pediatrics 92, 529–534 (1978). 305471 10.1016/s0022-3476(78)80282-0

[b28] Łeski J. M. & Henzel N. Ecg baseline wander and powerline interference reduction using nonlinear filter bank. Signal processing 85, 781–793 (2005).

[b29] R Core Team. R: A Language and Environment for Statistical Computing. R Foundation for Statistical Computing, Vienna, Austria, https://www.R-project.org/ (2016).

